# A multicenter study of modified electron beam output calibration

**DOI:** 10.1002/acm2.14232

**Published:** 2023-12-13

**Authors:** Dwi Aprilia Mahfirotin, Brian Ferliano, Andrian Dede Handika, Yosi Sudarsi Asril, Muhamad Fadli, Dea Ryangga, Nelly Nelly, Eddy Kurniawan, Wahyu Edy Wibowo, Poonam Yadav, Supriyanto Ardjo Pawiro

**Affiliations:** ^1^ Department of Physics Faculty of Mathematics and Natural Sciences Universitas Indonesia, Depok West Java Indonesia; ^2^ Department of Radiation Oncology Mitra Keluarga Bekasi Timur Hospital, Bekasi West Java Indonesia; ^3^ Department of Radiation Oncology Gading Pluit Hospital Jakarta Indonesia; ^4^ Department of Radiation Oncology Persahabatan Central General Hospital Jakarta Indonesia; ^5^ Department of Radiation Oncology Mayapada Hospital Jakarta Selatan Jakarta Indonesia; ^6^ Department of Radiation Oncology MRCCC Siloam Hospital Semanggi Jakarta Indonesia; ^7^ Department of Radiation Oncology Pasar Minggu Regional Hospital Jakarta Indonesia; ^8^ Department of Radiation Oncology Siloam Hospital TB Simatupang Jakarta Indonesia; ^9^ Department of Radiation Oncology Tzu Chi Hospital Jakarta Indonesia; ^10^ Department of Radiation Oncology Dr. Cipto Mangunkusumo National General Hospital Central Faculty of Medicine Universitas Indonesia Jakarta Indonesia; ^11^ Department of Radiation Oncology Northwestern Memorial Hospital Northwestern University Feinberg School of Medicine Chicago Illinois USA

**Keywords:** beam quality conversion factor, electron reference dosimetry, modified calibration, Monte Carlo simulation, TRS‐398 protocol

## Abstract

**Purpose:**

This study aims to assess the accuracy of a modified electron beam calibration based on the IAEA TRS‐398 and AAPM‐TG‐51 in multicenter radiotherapy.

**Methods:**

This study was performed using the Elekta and Varian Linear Accelerator electron beams with energies of 4–22 MeV under reference conditions using cylindrical (PTW 30013, IBA FC65‐G, and IBA FC65‐P) and parallel‐plate (PTW 34045, PTW 34001, and IBA PPC‐40) chambers. The modified calibration used a cylindrical chamber and an updated $k{{}^{\prime }}_{Q}$ based on Monte Carlo calculations, whereas TRS‐398 and TG‐51 used cylindrical and parallel‐plate chambers for reference dosimetry. The dose ratio of the modified calibration procedure, TRS‐398 and TG‐51 were obtained by comparing the dose at the maximum depth of the modified calibration to TRS‐398 and TG‐51.

**Results:**

The study found that all cylindrical chambers’ beam quality conversion factors determined with the modified calibration $\left({k^{\prime }}_{Q}\right)$ to the TRS‐398 and TG‐51 vary from 0.994 to 1.003 and 1.000 to 1.010, respectively. The dose ratio of modified/TRS‐398_cyl_ and modified/TRS‐398_parallel‐plate,_ the variation ranges were 0.980–1.014 and 0.981–1.019, while for the counterpart modified/TG‐51_cyl_ was found varying between 0.991 and 1.017 and the ratio of modified/TG‐51_parallel‐plate_ varied in the range of 0.981–1.019.

**Conclusion:**

This multi‐institutional study analyzed a modified calibration procedure utilizing new data for electron beam calibrations at multiple institutions and evaluated existing calibration protocols. Based on observed variations, the current calibration protocols should be updated with detailed metrics on the stability of linac components.

## 1 INTRODUCTION

The American Association of Physicists in Medicine (AAPM) and the International Atomic Energy Agency (IAEA) published protocols for clinical reference dosimetry in external beam radiation therapy using high‐energy photon and electron beams.^1^, ^2^ The protocols are AAPM Task Group 51 (TG‐51) and IAEA Technical Report Series‐398 (TRS‐398), published in 1999 and 2000, respectively. The formalism and dosimetry procedures in the TG‐51 and TRS‐398 protocols are based on the using an ionization chamber with a ^60^Co absorbed dose to water calibration factor $N_{D,w}^{60Co}$ and a beam quality conversion factor $k_{Q}$ for the user's beam. There are ongoing efforts to update both protocols. For instance, the Addendum to the AAPM TG‐51 protocol was published for reference dosimetry for high‐energy photon beams in 2014.^3^ Therefore, measuring reference dosimetry in electron beams also requires further revision.

For electron beams, the beam quality for TG‐51 and TRS‐398 is specified by the depth of the 50% absorbed dose in water, *R*_50_. Clinical reference dosimetry is performed at a depth $z_{ref}\;=\;0.6R_{50}-0.1\;$(cm), obtained from *R*_50_. The beam quality conversion factors $k_{R_{50}}$ and $k_{ecal},\;$gradient correction factor $P_{gr}^{Q}$, and percentage depth dose at the reference depth, PDD$\left(z_{ref}\right)$, need to be determined in TG‐51. In contrast, TRS‐398 uses $k_{Q,Qo}$ instead of $k{{}^{\prime }}_{R_{50}}$ and $k_{ecal}$ in TG‐51.

Several international dosimetry protocols^1^, ^2^ recommend using parallel‐plate chambers for electron beam measurements, especially for low‐energy electrons ($R_{50}<$4 g/cm^2^ or below 10 MeV). Electron beam measurement using a parallel‐plate chamber can effectively minimize fluence perturbation^4^ as the front windows of a parallel‐plate chamber are thin and composed of materials with similar properties to water; therefore, the effect of the wall can be neglected.^5^ Some protocols do not recommend using a cylindrical chamber for electron beam dosimetry at low energies.^1^, ^2^ This is because a cylindrical chamber can have a large fluence correction factor (up to 5%), which could be a source of high uncertainty.^6^ A more recent publication^7^ states that the variability in perturbation corrections for cylindrical chambers is 0.4%, and the results are not significantly different from those for parallel‐plate chambers with the same specifications. They proposed a cylindrical chamber for reference dosimetry of all electron beams. Additionally, literature has shown superior (always < 0.15%) long‐term stability of a cylindrical chamber in an electron beam than parallel‐plate chambers (0.2−0.4%).^8^

Determining $k_{Q}$ recommended in the current dosimetry protocol requires several assumptions in electron beams. For example, the wall correction factor in the electron beam was assumed to be unity, and in some cases, some data exhibit a systematic uncertainty.^9^ The beam quality conversion factor, $k_{Q}$, based on the protocol has been shown to have a nearly 2% difference compared to the more accurate Monte Carlo calculations.^10^

In view of existing challenges, Muir and Rogers^11^ studied beam quality conversion factors using Monte Carlo calculations (EGSnrc code system)^12^ with the PTW Roos and NE2571 chambers. A year later, extensive research was conducted with 18 cylindrical and 10 parallel‐plate chambers. The most complete set of electron beam quality conversion factor datasets for multiple chambers was obtained.^9^ In the Monte Carlo calculations, the beam quality conversion factor was introduced with a new notation as a subscript *Q* rather than *R*_50_. This clarifies that the gradient corrections were implicitly calculated and included. Two studies by Erazo^13^, ^14^ provide Monte Carlo calculated data for cylindrical chambers and other studies^15^, ^16^ focused on the beam quality factor $k_{Q\;}$calculated for parallel‐plate chambers. The systematic uncertainties in calculated beam quality conversion factors for the NE2571 chambers are less than 1.2% using conservative assumptions.^11^ The $p_{wall}$ correction factor calculated for the reference beam quality Co‐60 is in good agreement with the Monte Carlo based data published in recent years.^11^ The overall uncertainty of calculated beam quality conversion factors was estimated to be <0.7%.^15^ Due to different positioning recommendations in the international dosimetry protocols, the uncertainty in the range is 0.2%–0.6%. Thus, this work may reduce the uncertainty significantly.

Based on specific considerations, Muir et al. proposed a new equation to calculate the absorbed dose in an electron beam without using the gradient correction factor by the clinical physicist.^17^ Their work suggested that the cylindrical chamber could be used as reference dosimetry for all electron beam energies to make electron beam dosimetry measurements simpler and easier to perform by aligning the procedure with that for photon beams.

The importance of achieving high accuracy and precision in radiotherapy has always been recognized, and dosimetry plays a crucial role in achieving this goal. Pawiro et al.^18^ recently compared the absolute dose ratio using the modified calibration procedure and TRS‐398 protocols. The results showed that the absolute dose ratios obtained with the cylindrical chamber were 1.002 and 1.004. Yulinar et al.^19^ also carried out absolute dose ratio using modified calibration procedure compared to TRS‐398 dan TG‐51 protocols. The average absolute dose ratio of modified/TRS‐398 and modified/TG‐51 were 1.004 and 1.009, respectively. The results were below the tolerance limit (±2%) based on IAEA TRS‐398, with a deviation of 0.9%–1.06% for the modified calibration.

The purpose of this study is to apply a modified calibration procedure for electron beam calibrations then compare the results to those obtained using existing calibration protocols, which are TRS‐398 and TG51 protocols at multiple institutions. Dose determined using cylindrical and parallel‐plate chambers according to the modified calibration were compared against the existing IAEA TRS‐398 and AAPM TG‐51 protocols.

## 2 MATERIALS AND METHODS

### 2.1 Dosimetry equipment and experimental measurement

Experimental measurements were conducted using a set of 14 ionization chambers consisting of two PTW 30013 (Farmer), four IBA FC65‐G (Scanditronix / Wellhofer Farmer), and an IBA FC65‐P (Scanditronix / Wellhofer Farmer) type cylindrical chambers, and a PTW 34045 (Advanced Markus), PTW 34001 (Roos), and five IBA PPC‐40 (Scanditronix / Wellhofer) type parallel‐plate chambers. All the cylindrical and parallel‐plate chambers and their characteristics are listed in Tables 1 and 2, respectively. The absolute charge was measured using the electrometer models UNIDOS and Tandem (PTW, Germany) and Dose‐1 (IBA, GmBH, Scanditronix Wellhofer, Germany). Two PTW 30013 chambers were connected to the UNIDOS and Tandem electrometer, and the PTW 34045, PTW 34001, IBA FC65‐G, IBA FC65‐P, and IBA PPC‐40 chambers were connected to the Dose‐1 electrometer. The operating voltages for the PTW 30013 chamber were ±400 and 100 V, whereas those for the PTW 34045, PTW 34001, IBA FC65‐G, IBA FC65‐P, and IBA PPC‐40 chambers were ±300and 100 V. The operational voltage with two‐voltage method is used for measurement of ion recombination correction factor, $P_{ion}.$

**TABLE 1:** The characteristics of cylindrical chamber^2^ used in this study.

	Cavity wall			
Ionization chamber	Material	Thickness (g/cm^2^)	Cavity volume (cm^3^)	Waterproof (Y/N)
PTW 30013	PMMA	0.057	0.6	Y
IBA FC65‐G	Graphite	0.073	0.65	Y
IBA FC65‐P	Polyoxymethylene	0.057	0.65	Y

**TABLE 2:** The characteristics of parallel‐plate chamber^2^ used in this study.

	Entrance window		
Ionization chamber	Material	Thickness (g/cm^2^)	Waterproof (Y/N)
PTW 34045	Graphite, polyethylene	0.102	Y
PTW 34001	PMMA, graphite	0.118	Y
IBA PPC‐40	PMMA, graphite	0.118	Y

An Indonesian secondary standard dosimetry laboratory (National Nuclear Energy Agency of Indonesia) provided the absorbed dose to water calibration factor $N_{D,w}^{60Co}$ for the 14 ionization chambers. The chamber types used at each center, electrometer models, and the model of water phantoms are listed in Table 3.

**TABLE 3:** The chamber types, electrometer models, and the model of water tank.

Center	Ionization chamber	Electrometer model	Water phantom model
1	PTW 30013	PTW Tandem	PTW Beam Scan Lift
	PTW 34045		
2	PTW 30013	PTW Unidos	Sun Nuclear 3D Scanner
	PTW 34001		
3	IBA FC65‐G	IBA Dose 1	IBA Water tank 1D
	IBA PPC‐40		
4	IBA FC65‐G	IBA Dose 1	IBA Water tank 1D
	IBA PPC‐40		
5	IBA FC65‐G	IBA Dose 1	Sun Nuclear 1D Scanner
	IBA PPC‐40		
6	IBA FC65‐G	IBA Dose 1	IBA Water tank 1D
	IBA PPC‐40		
7	IBA FC65‐P	IBA Dose 1	IBA Water tank 1D
	IBA PPC‐40		

The measurement of absorbed dose to water was performed at seven radiotherapy centers for seven clinical electron beams delivered by four Elekta medical linear accelerator types, a Synergy Platform linear accelerator, a Precise linear accelerator, and two Versa HD linear accelerators, as well as by three Varian medical linear accelerator types, a Clinac iX linear accelerator, and two Trilogy linear accelerators. The electron beams used were in the energy range of 4−22 MeV, depending on the energy in each radiotherapy center. The linear accelerators were operated at a dose rate of 100 MU/min. A 10 × 10 cm^2^ applicator was used to shape the electron beam field. The gantry and collimator angles were set to zero degrees. The measurements were repeated three times.

In TRS‐398, the position of the reference point for the parallel‐plate chamber was at the reference depth ($z_{ref}),\;$and on the inner surface of the entrance window, at the center of the window, whereas for the cylindrical chamber, it was at the effective point of measurement, 0.5 $r_{cyl}\;$deeper than $z_{ref}$. This is different for measurements using modified calibration procedure by Muir^17^ and Pawiro et al.,^18^ where the position of the reference point for the cylindrical chamber was with the central axis at $z_{ref}.$ The measurement of $z_{ref}$ in the absolute dosimetry of the electron beam is calculated as follows:

(1) $$Z_{ref}=\;0.6R_{50}-0.1\;\left(\mathrm{cm}\right),$$

where $R_{50}\;$is the depth at which dose falls 50% of the maximum for 10 cm × 10 cm field size. The types of linear accelerators, $R_{50},\;$and $z_{ref}$ used in the measurements are listed in Table 4.

**TABLE 4:** Characteristics of the electrons beams used.

Center	Linear accelerator	Energy (MeV)	R_50_ (cm)	$z_{ref}$ (cm)
1	Elekta Synergy Platform	6	2.46	1.38
		8	3.20	1.82
		10	3.94	2.26
		12	4.67	2.70
		18	7.09	4.15
2	Elekta Precise	4	1.70	0.92
		6	2.48	1.39
		8	3.25	1.85
		10	3.94	2.26
		15	5.90	3.43
		18	7.05	4.13
3	Elekta Versa HD	6	2.51	1.41
		9	3.65	2.09
		12	4.76	2.76
4	Varian Clinac iX	6	2.38	1.33
		9	3.63	2.08
		12	5.08	2.95
		16	6.72	3.93
		20	8.38	4.93
5	Varian Trilogy	6	2.39	1.33
		9	3.54	2.02
		12	4.98	2.89
		18	7.49	4.39
		22	8.69	5.11
6	Varian Trilogy	6	2.40	1.34
		9	3.59	2.05
		12	5.03	2.92
		15	6.35	3.71
		18	7.61	4.47
7	Elekta Versa HD	4	1.73	0.94
		6	2.52	1.41
		8	3.23	1.84
		10	3.97	2.28
		12	4.78	2.77
		15	6.07	3.54

### 2.2 Determination of the beam quality conversion factor and absorbed dose to water

The radiation beam quality index (*Q*) is an important parameter for determining the beam quality conversion factor. Based on TRS‐398, the beam quality conversion factor, denoted as $k_{Q,Qo}$, was determined by interpolating the *R*_50_ data in Table 5 of the TRS‐398 protocol.^2^ Beam quality conversion factor, $k_{Q,Qo}$, of the IBA FC65‐G and IBA FC65‐P cylindrical chambers was obtained using data for the Scanditronix—Wellhofer FC65‐G and Scanditronix—Wellhofer FC65‐P data chambers, respectively. The $k_{Q,Qo}$ of PTW 34045 was using Advanced Markus chambers data, whereas PTW 34001, and IBA PPC‐40 parallel‐plate chambers was found using Roos chamber data. Beam quality conversion factor, $k_{Q,Qo}$, is needed to calculate the absorbed dose to water $D_{w,Q}$, as shown in Equation (2).

(2) $$D_{w,Qo\;\left(Zref\right)}=\;M_{Qo}\;N_{D,w,Qo}\;k_{Q,Qo},$$

where $M_{Qo}\;$is the fully corrected charge reading from an ionization chamber in a water phantom (nC),$\;N_{D,w,Qo}\;$is the absorbed dose to water calibration factor for the ^60^Co beam of each chamber (Gy/nC), and $k_{Q,Qo}$ is the beam quality conversion factor.

**TABLE 5:** Calculated values of $k_{Q}$ for electron beams in various chamber types, as a function of beam quality *R*_50_.^2^

	Beam quality R_50_ (g/cm^2^)																
Ionization chamber type	1.0	1.4	2.0	2.5	3.0	3.5	4.0	4.5	5.0	5.5	6.0	7.0	8.0	10.0	13.0	16.0	20.0
Parallel‐plate chambers																	
Markus	–	–	0.925	0.920	0.916	0.913	0.910	0.907	0.904	0.901	0.899	0.894	0.889	0.881	0.870	0.860	0.849
Roos	0.965	0.955	0.944	0.937	0.931	0.925	0.920	0.916	0.912	0.908	0.904	0.898	0.892	0.882	0.870	0.860	0.848
Cylindrical chambers																	
PTW 30013 (Farmer)	–	–	–	–	–	–	0.911	0.909	0.907	0.906	0.904	0.901	0.898	0.893	0.885	0.878	0.868
Scdx‐Wellhofer FC65‐P	–	–	–	–	–	–	0.914	0.912	0.911	0.909	0.907	0.904	0.902	0.896	0.889	0.881	0.872
Scdx‐Wellhofer FC65‐G	–	–	–	–	–	–	0.920	0.918	0.916	0.914	0.913	0.910	0.907	0.902	0.894	0.887	0.877

The determination of the beam quality conversion factor in the modified calibration, denoted as $k{{}^{\prime }}_{Q}$, was the same as that of Pawiro^18^ and Muir's work.^17^ The factor of $k{{}^{\prime }}_{Q}$ of a chamber is determined by substituting the power fitting parameter for a cylindrical chamber to the equation based on the Monte Carlo calculation proposed by Muir and Rogers.^9^ Factor of $k{{}^{\prime }}_{Q}$, can be used to calculate the absorbed dose to water at depth reference $D_{w,Q(Zref)}$, as in the following equation:

(3) $$D_{w,Q\left(Zref\right)\;}=\;M\;k{{}^{\prime }}_{Q}\;k_{Q,ecal}\;N_{D,w}^{co}$$

where *M* is the ion chamber measurement in the water phantom (corrected for temperature, pressure, polarity, ion recombination, and electrometer response). The factor of $k{{}^{\prime }}_{Q}$ is the beam quality conversion factor without using an effective point of measurement and including the gradient effects by definition. The factor of $k_{Q,ecal}$ is the photon–electron conversion factor. The factor of $k_{Q,ecal}$ was chamber specific, with 0.901 for PTW 30013, 0.904 for IBA FC65‐G, and 0.902 for IBA FC65‐P^9^ and $N_{D,w}^{co}$ is the Cobalt‐60 absorbed dose calibration coefficient.

All dose measurements at the reference depth $\left(z_{ref}\right)$ were converted to the dose at the maximum depth $\left(z_{max}\right)$ following equation:

(4) $$D_{w,Q}\;\;\left(z_{max}\right)=\frac{100\;\times D_{w,Q}\left(z_{ref}\right)}{\;(PDD_{\left(Zref\right)}\;)}\;.$$

### 2.3 Analysis of the data

The absorbed dose at a maximum depth using the modified calibration methods and TRS‐398 using cylindrical and parallel‐plate chambers can be obtained according to Equation (4). We then compared the absorbed dose determined with the modified calibration method, TRS‐398, and TG‐51 protocols. The dose ratios between modified calibration to TRS‐398 using a parallel‐plate chamber, modified calibration to TRS‐398 using a cylindrical chamber, modified calibration to TG‐51 using a parallel‐plate chamber, and modified calibration to TG‐51 using a cylindrical chamber were analyzed. The dose ratio of modified calibration to TRS‐398 using cylindrical chambers is denoted as modified/TRS‐398_cyl._ The dose ratio of modified calibration to TRS‐398 using a parallel‐plate chamber is denoted as modified/TRS‐398_parallel‐plate._ The dose ratio of modified calibration to TG‐51 using cylindrical chambers is denoted as modified/TG‐51_cyl_. The dose ratio of modified to TRS‐398 using a parallel‐plate chamber is denoted as modified/TG‐51_parallel‐plate_. The analysis was performed based on the dose ratio and difference.

## 3 RESULTS

**FIGURE 1:**
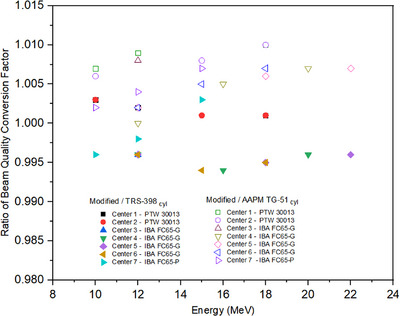
The ratio of beam quality conversion factors of cylindrical chamber using modified calibration to TRS‐398 and TG‐51.

**FIGURE 2:**
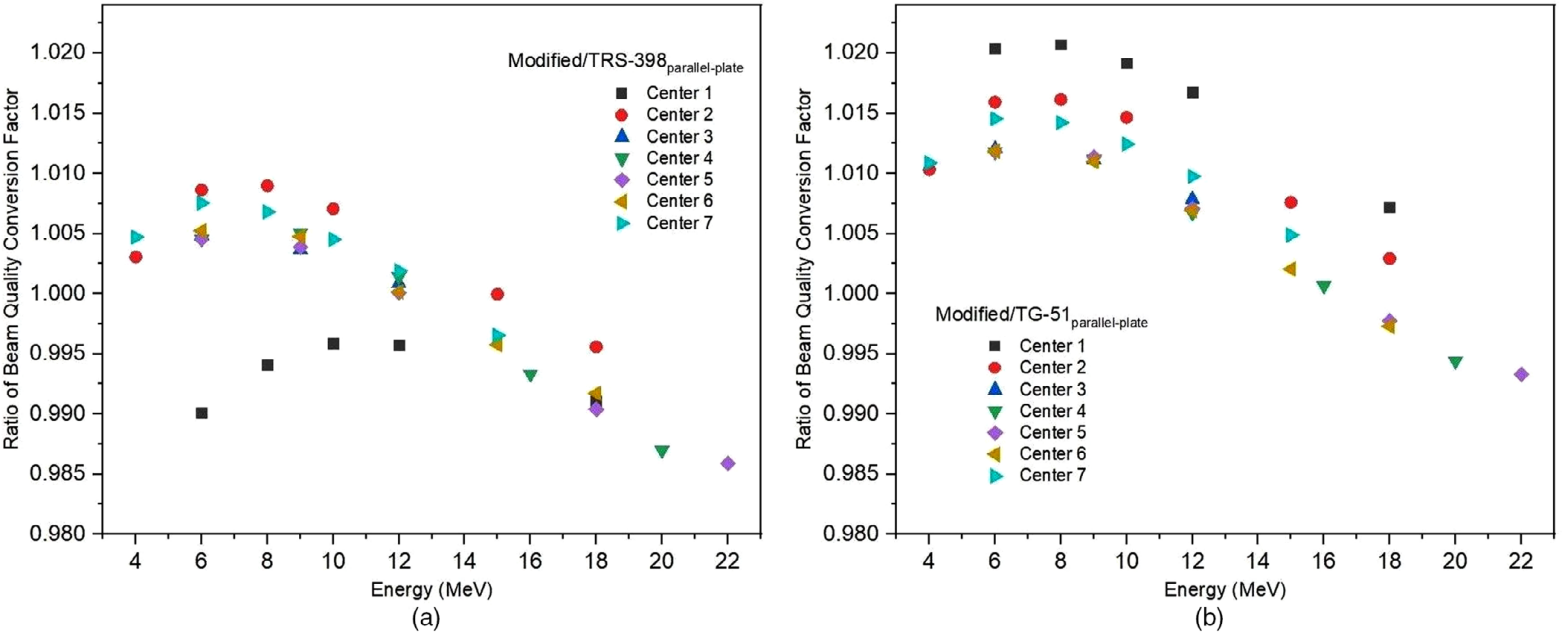
The ratio of beam quality conversion factors of parallel‐plate chambers using (a) modified calibration to TRS‐398 and (b) modified calibration to TG‐51.

The beam quality conversion factors for several ionization cylindrical chambers and parallel‐plate chambers are presented in Figures 1 and 2, respectively. Figure 1 shows the ratio of beam quality conversion factors of cylindrical chambers using modified calibration to TRS‐398 and TG‐51. In the modified calibration procedure, the beam quality conversion factor of cylindrical chamber could be determined for all energy beams. This is different where the beam quality conversion factor of the cylindrical chamber for TRS‐398 and TG‐51 could not be determined at low energies (E< 10 MeV). Therefore, the plot in Figure 1 only shows the results of ratio beam quality conversion factor in high energy electron beam (10 MeV ≥E≤ 22 MeV). The ratio beam quality conversion factor, $k_{Q,Qo}$ in high‐energy electron beam of modified/TRS‐398cyl varied from 0.994 to 1.003 while modified/TG‐51cyl varied from 1.000 to 1.010.

According to Figure 2, the beam quality conversion factor, $k_{Q,Qo}\;$of the parallel‐plate chamber could be determined for all electron energy beams using TRS‐398 and TG‐51. Figure 2(a) show the ratio of beam quality conversion factor of modified to TRS‐398 and Figure 2(b) show the ratio of beam quality conversion factor of modified to TG‐51. The ratio of beam quality conversion factor in Figure 2(a) is in the range of 0.986–1.009 while the ratio of beam quality conversion factor in Figure 2(b) is in the range of 0.993–1.021 and hence implies that the ratio of beam quality conversion factor of modified calibration to TRS‐398 and TG‐51 in the range of ±2%.

**FIGURE 3:**
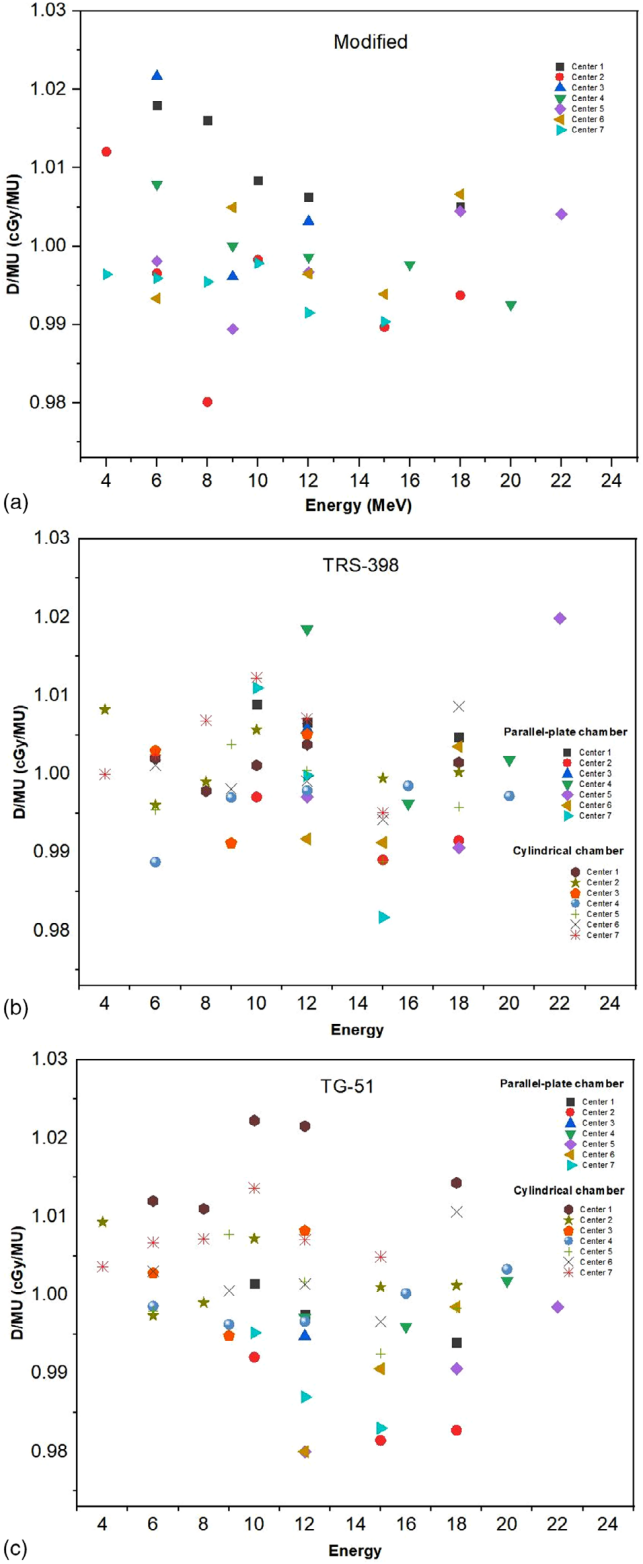
The dose per monitor unit obtained by three methods (a) modified calibration, (b) TRS‐398 and (c) TG‐51.

The main results of this study are shown in Figure 3. Figure 3(a) shows a results of dose per monitor unit using modified calibration procedure in each center of radiotherapy. In this study, the results of modified procedure are obtained only by cylindrical chamber. The results for dose/MU are varying from 0.980 to 1.022 cGy/MU. Figure 3(b) show results of dose per monitor unit obtained by TRS‐398 using cylindrical chamber and parallel‐plate chamber. The dose per monitor unit in low energy beams (E ≤ 10 MeV) is only the contribution from the data for parallel‐plate chamber. The results of dose/MU are varying from 0.982 to 1.020 cGy/MU and 0.989 to 1.012 cGy/MU for cylindrical chamber and parallel‐plate chambers, respectively. Figure 3(c) show a results of dose per monitor unit obtained by TG‐51 using cylindrical and parallel‐plate chamber. Cylindrical chambers are only applied at low energy beams. The results of dose/MU are varying from 0.980 to 1.002 cGy/MU and 0.993 to 1.022 cGy/MU for cylindrical chamber and parallel‐plate chambers, respectively.

The results of dose per monitor unit of modified calibration procedure are compared to the TRS‐398 and TG‐51 to determine the consistency in the level accuracy of the modified calibration procedure. Table 6 shows the dose ratio of modified calibration to TRS‐398 and TG‐51 protocols. The dose ratio of modified/TRS‐398_cyl_ and modified/TG‐51_cyl_ could only be calculated for high‐energy beams (E≥ 10 MeV). This is because TRS‐398 and TG‐51 recommend using a cylindrical chamber at high energy (*R*_50_ ≥4 g cm^−2^) for electron beam calibration.^2^ Therefore, the dose determined using the modified calibration method at low energy (E < 10 MeV) could not be compared to that of TRS‐398 using a cylindrical chamber. Based on Table 6, the dose ratio of modified/TRS‐398_cyl_ at Centers 1, 2, 3, 4, 5, 6, and 7 resulted in minimum and maximum value of 0.980–1.014, while the dose ratio of modified/TG‐51_cyl_ resulted in 0.991–1.017. The dose ratio of modified/TRS‐398_parallel‐plate_ has resulted in the range of 0.981–1.019, while the dose ratio of modified/TG‐51_parallel‐plate_ has resulted in the range of 0.981–1.019. Overall, its mean that the absolute dose obtained by modified calibration procedure is in the range of tolerance limit of ±2%.^2^

**TABLE 6:** Dose ratio between the modified calibration and TRS‐398 using a cylindrical chamber (modified/TRS‐398_cyl_), and a parallel‐plate chamber (modified/TRS‐398_parallel‐plate_), modified calibration and TG‐51 using a cylindrical chamber (modified/TG‐51_cyl_), and using a parallel‐plate chamber (modified/TG‐51_parallel‐plate_) in multicenter radiotherapy.

Center	Energy (MeV)	Modified/ TRS‐398_cyl_	Modified/ TRS‐398_parallel‐plate_	Modified/ TG‐51_cyl_	Modified/ TG‐51_parallel‐plate_
1	6	–	1.016	–	1.006
	8	–	1.018	–	1.005
	10	0.999	1.007	1.007	0.986
	12	1.000	1.002	1.009	0.985
	18	1.000	1.004	1.011	0.991
2	4	–	1.004	–	1.003
	6	–	1.000	–	0.999
	8	–	0.981	–	0.981
	10	1.001	0.993	1.006	0.991
	15	1.001	0.990	1.008	0.989
	18	1.002	0.994	1.011	0.993
3	6	–	1.019	–	1.019
	9	–	1.005	–	1.001
	12	0.998	0.998	1.008	0.995
4	6	–	1.019	–	1.009
	9	–	1.003	–	1.004
	12	0.980	1.001	1.001	1.002
	16	1.001	0.999	1.001	0.997
	20	0.991	0.995	0.991	0.989
5	6	–	1.003	–	1.000
	9	–	0.986	–	0.982
	12	1.000	0.996	1.017	0.995
	18	1.014	1.016	1.014	1.012
	22	0.984	1.008	1.006	1.006
6	6	–	0.992	–	0.990
	9	–	1.007	–	1.004
	12	1.005	0.997	1.017	0.995
	15	1.003	1.000	1.003	0.997
	18	1.003	0.99	1.008	0.996
7	4	–	0.996	–	1.000
	6	–	0.994	–	1.002
	8	–	0.989	–	1.007
	10	0.987	0.986	1.003	1.012
	12	0.992	0.985	1.005	1.007
	15	1.009	0.995	1.007	0.995

Estimating the uncertainty of measurements obtained through a modified calibration method is crucial to ensure accurate results. This step is integral to the measurement process and must be noticed. By taking into account the potential sources of error and quantifying their impact, we have confidence in the validity of our findings. The standard uncertainties are classified into A and B types. Type A standard uncertainty is obtained from a series of repeated observations, and type B standard uncertainty is evaluated by methods other than a statistical analysis of a series of observations. The uncertainty budget of absorbed dose to water determined at maximum depth ($z_{max})$ under reference conditions using a modified calibration method is shown in Table 6. This work estimated the component that took in this measurement are SSD setting, $P_{TP}$ correction, $P_{ion}\;$correction, $P_{pol}\;$correction, $P_{elec}\;$correction, humidity, charge measurement, corrected reading, calibration factor $\left(N_{D,w}\right)$, and beam quality conversion factor ($k_{Q,Qo})$. According to Table 7, the results show a typical uncertainty in determining the absorbed dose of water using modified electron beam calibration of approximately 0.54%.

**TABLE 7:** Estimated uncertainty budgets of absorbed dose to water that determined at maximum depth ($z_{max})$ under reference conditions using modified calibration method.

Component	Uncertainty (%)
SSD setting	0.10
Positioning the chamber	0.17
Field size setting	0.10
$P_{TP}$ correction	0.10
$P_{ion}\;$correction	0.10
$P_{pol}\;$correction	0.05
$P_{elec}\;$correction	0.07
Humidity	0.05
Charge measurement	0.23
Beam quality conversion factor ($k_{Q,Qo})$	0.40
Total uncertainty	0.54

## 4 DISCUSSION

### 4.1 Beam quality conversion factor for modified calibration procedure to the TRS‐398 and TG‐51

The results of ratio beam quality conversion factor of modified compared to TRS‐398 and TG‐51 are shown in Figures 1 and 2. The minimum and maximum difference in beam quality conversion factor of modified to TRS‐398 was 0.05%, and −0.6%, respectively. This value is according to the results of ratio beam quality conversion factor in high energy electrons beam (10 MeV ≥E≤ 22 MeV), which lowest value of 1.001 to the highest of 0.994. While the difference of beam quality conversion factor ratio of the modified for TG‐51 was 0.14%% and 1.04%. The beam quality conversion factor of cylindrical chamber using modified was also compared to the parallel plate chamber using TRS‐398 and TG‐51. The ratio beam quality conversion factor of modified to TRS‐398 was from 0.00 to −1.41%, while the ratio beam quality conversion factor of modified to TG‐51 was from 0.07% to 2%. The factor $k{{}^{\prime }}_{Q}$ in the modified calibration using Monte Carlo calculation incorporated detailed information about the ionization chamber to better reflect the actual geometry.^20^ Monte Carlo calculated $k{{}^{\prime }}_{Q}$ factors should be more accurate because ionization chambers can be reliably modelled and, the Monte Carlo algorithms have been shown to be accurate. Monte Carlo calculations can show inaccuracies in determining some perturbation factors, such as $p_{wall}$ and $p_{dis}$. The $p_{wall}$ factor explains the non‐water equivalence of the ionization chamber wall, whereas $p_{dis}$ shows the effect due to the replacement of water by the air cavity.^21^ The determination of the beam quality conversion factor with high accuracy and investigation of its influencing quantities are important to reduce the uncertainties of dose measurement. However, $k_{Q}$ values depend on the design and size of the ionization chamber, as well as on the materials of the chamber components.^22^

In contrast, the results for TRS‐398 were based on a semi‐analytic approach that does not consider all the details of the ionization chamber geometry.^2^ According to cavity theory,^23^ $k_{Q,Qo}$ in TRS‐398 is expressed in terms of the Spencer–Attix water‐to‐air stopping power ratios and several perturbation factors. However, the validity of $k_{Q,Qo}$ based on this cavity theory implies a number of estimates, such as the independence of the perturbation factor and the lack of correlation between stopping power ratios and the perturbation effect. Several studies^19^, ^24^ have compared the perturbation correction factor obtained using Monte Carlo calculations on a cylindrical chamber with the results obtained using a semi‐analytic approach with TRS‐398. Therefore, the difference in calculating the beam quality conversion factor obtained between the two methods was due to the difference inherent to the semi‐analytic approach and Monte Carlo calculation.

### 4.2 The dose ratio between modified calibration procedure to TRS‐398 and TG‐51 protocols (modified/TRS‐398) and (modified/TG‐51)

It can be seen from Table 6 that there is a component that contributes to the differences in absorbed dose to water between modified calibration and TRS‐398. This uncertainty in the charge reading measurement procedure is common to the modified calibration and TRS‐398 protocol. The dose ratio of modified calibration procedure to the TRS‐398_cyl_ has the deviation between −2% and 0%, while the dose ratio of modified calibration procedure to the TG‐51_cyl_ has the deviation between −0.9% and 1.7%. The dose ratio of modified calibration procedure to TRS‐398_parallel‐plate_ has the deviation between −1.89% and 1.9%, while the dose ratio of modified calibration procedure to the TG‐51_parallel‐plate_ has the deviation between −1.9% and 1.9%. Overall, the deviation obtained by modified calibration procedure are in the range of ±2%. Thus, this modified calibration procedure potentially uses a cylindrical chamber for electron beam calibration in all energy beams.

The cylindrical chamber was compared with the parallel plate chamber in low electron energy beams (4 ⩽ E < 10 MeV). For low‐energy beams, the calculation of $k_{Q}$ is highly sensitive to the ionization chamber model. Therefore, $k_{Q}$ in the calculation of absorbed dose electrons at low electron energy beams contributes to the high uncertainty.^11^, ^15^ The difference of the dose ratio in parallel‐plate chambers is due to the chamber material composition, chamber geometry and beam quality conversion factor. The difference in the dose ratio in cylindrical ionization chambers is due to the effect of volume averaging on chamber response, and it should be noted that all experimental determinations entering into $k_{Q}$ are corrected for volume averaging (either explicitly or implicitly).^25^ Another potential cause is the influence of the ionization chamber position, which can affect the reading (M) at $z_{ref}$. The cylindrical chamber with modified calibration was placed on the central axis of $z_{ref}$. In contrast for the TRS‐398 protocol, it was placed 0.5 *r_cyl_* deeper than the reference depth ($z_{ref})$. This was investigated in another study^26^ where the value of $D_{ch}\left(z_{ref}\right)$ was compared with $D_{ch}(z_{ref}\pm$0.5 mm) in the NE2571, Exradin A1SL, and PTW Roos chambers. The uncertainty for low electron energy beams was higher, approximately 0.05%−0.35%, whereas it was always less than 0.2% for high electron energy beams. The position of the ionization chamber influences the readings of the ionization chamber, although there is a slight dependence.

## 5 CONCLUSION

The study found that all cylindrical chambers’ beam quality conversion factor determined with the modified calibration $\left({k^{\prime }}_{Q}\right)$ when compared to the TRS‐398 and TG‐51 vary from 0.994 to 1.003 and 1.000 to 1.010, respectively. The dose ratio of modified/TRS‐398_cyl_ and modified/TRS‐398_parallel‐plate,_ are in 0.980–1.014 and 0.981–1.019 range, while for the counterpart modified/TG‐51_cyl_ was found vary between 0.991 and 1.017 and the ratio of modified/TG‐51_parallel‐plate_ was vary in the range of 0.981–1.019. In conclusion, a modified calibration procedure can be used for the electron beam if different energies are used for reference dosimetry.

## AUTHOR CONTRIBUTIONS

Dwi A. Mahfirotin was responsible in research design, data collection, data analysis and manuscript writing. Brian Ferliano contributed in data collection. Andrian Dede Handika contributed in data collection. Yosi Sudarsi Asril contributed in data collection. Muhamad Fadli contributed in data collection. Dea Ryangga contributed in data collection. Nelly Nelly contributed in data collection. Eddy Kurniawan contributed in data collection. Wahyu E. Wibowo contributed in data collection. Poonam Yadav contributed in manuscript writing and data analysis. Supriyanto A. Pawiro was responsible in data collection, data analysis, and manuscript writing.

## CONFLICT OF INTEREST STATEMENT

The authors declare no potential conflicts of interest.

## References

[1] Almond PR , Biggs PJ , Coursey BM , et al. AAPM's TG–51 protocol for clinical reference dosimetry of high‐energy photon and electron beams. Med Phys. 1999;26:1847‐1870. 10505874 10.1118/1.598691

[2] Andreo P , Burns DT , Hohlfeld K , et al. Absorbed dose determination in external beam radiotherapy: An International Code of Practice for Dosimetry Based on Standards of Absorbed Dose to Water: Technical Reports Series No. 398. International Atomic Energy Agency; 2000.

[3] McEwen M , DeWerd L , Ibbott G , et al. Addendum to the AAPM's TG‐51 protocol for clinical reference dosimetry of high energy photon beams. Med Phys. 2014;41(4):041501. 24694120 10.1118/1.4866223 PMC5148035

[4] Burns DT , McEwen MR . Ion recombination correction for the NACP parallel‐plate chamber in a pulsed electron beam. Phys Med Biol. 1998;43:2033‐2045. 9725587 10.1088/0031-9155/43/8/003

[5] Zink K , Czarnecki D , Looe H , Voigts‐Rhetz P , Harder D . Monte Carlo study of the depth‐dependent fluence perturbation in parallel‐plate ionization chambers in electron beams. Med Phys. 2014;41:111707. 25370621 10.1118/1.4897389

[6] Wittkamper FW , Thierens H , Van Der Plaetsen A , et al. Perturbation correction factors for some ionization chambers commonly applied in electron beams. Med Phys. 1991;36:1639.

[7] Muir BR , McEwen MR . Technical note: on the use of cylindrical ionization chambers for electron beam reference dosimetry. Med Phys. 2017;44(12):6641‐6646. 28913919 10.1002/mp.12582

[8] Muir BR , Cojocaru CD , McEwen MR , CK Ross . Electron beam water calorimetry measurements to obtain beam quality conversion factors. Med Phys. 2017;44:5433‐5444. 28688120 10.1002/mp.12463

[9] Muir BR , Rogers DWO . Monte Carlo calculations of electron beam quality conversion factors for several ion chamber types. Med Phys. 2014;41:111701. 25370615 10.1118/1.4893915

[10] Buckley LA , Rogers DWO . Wall correction factors, Pwall for thimble ionization chambers. Med Phys. 2006;33(2):455‐464. 16532953 10.1118/1.2161403

[11] Muir BR , Rogers DWO . Monte Carlo calculations for reference dosimetry of electron beams with the PTW Roos and NE2571 ion chambers. Med Phys. 2013;40:121722. 24320508 10.1118/1.4829577

[12] Kawrakow I , Mainegra‐Hing E , Rogers DWO , et al. The EGSnrc Code System: Monte Carlo Simulation of Electron and Photon Transport. National Research Council Canada; 2011. NRCC Technical Report PIRS‐701 v4‐2‐3‐2.

[13] Erazo F , Lallena AM . Calculation of beam quality correction factors for various thimble ionization chambers using the Monte Carlo Code PENELOPE. Phys Med. 2013;29:163‐170. 22277185 10.1016/j.ejmp.2012.01.001

[14] Erazo F , Brualla L , Lallena AM . Computation of the electron beam quality kq,qo factors for the NE2571, NE2571A and NE2581A thimble ionization chambers using PENELOPE. Phys Med. 2017;38:76‐80. 28610700 10.1016/j.ejmp.2017.05.053

[15] Zink K , Wulff J . Monte Carlo calculations of beam quality conversion factors kQ for electron dosimetry with a parallel‐plate Roos Chamber. Phys Med Biol. 2008;53:1595‐1607. 18367790 10.1088/0031-9155/53/6/006

[16] Zink K , Wulff J . Beam quality correction for parallel‐plate ion chambers in electron reference dosimetry. Phys Med Biol. 2012;57(7):1831‐1854. 22411097 10.1088/0031-9155/57/7/1831

[17] Muir BR . A modified formalism for electron beam reference dosimetry to improve accuracy of linac output calibration. Med Phys. 2020;47(5):2267‐2276. 31985833 10.1002/mp.14048

[18] Pawiro SA , Mahfirotin DA , Assegab MI , Wibowo WE . Modified electron beam output calibration based on IAEA Technical Report Series 398. J Appl Clin Med Phys. 2022;23(4):e13573. 35226389 10.1002/acm2.13573 PMC8992941

[19] Yulinar C , Assegab MI , Wibowo WE , Pawiro SA . Modified calibration protocols in electron beam dosimetry: comparison with IAEA TRS‐398 and AAPM TG‐51. Biomed Phys Eng Express. 2023;9:055008. 10.1088/2057-1976/ace722 37442101

[20] Muir BR , Rogers DWO . Monte Carlo calculations of kQ, the beam quality conversion factor. Med Phys. 2010;37:5939‐5950. 21158307 10.1118/1.3495537

[21] Sempau J , Andreo P , Aldana J , et al. Electron beam quality conversion factors for plane‐parallel ionization chambers: Monte Carlo calculations using the PENELOPE system. Phys Med Biol. 2004;49:4427‐4444. 15509075 10.1088/0031-9155/49/18/016

[22] Alissa M , Zink K , Tessier F , et al. Monte Carlo calculated beam quality conversion factors for two cylindrical ionization chambers in photon beams. Phys Med. 2022;94:17‐23. 34972070 10.1016/j.ejmp.2021.12.012

[23] Attix FH . Introduction to Radiological Physics and Radiation Dosimetry. Wiley; 1986.

[24] Wulff J , Heverhagen JT , Zink K . Monte‐Carlo‐based perturbation and beam quality conversion factors for thimble ionization chambers in high‐energy photon beams. Phys Med Biol. 2008;53(11):2823‐2836. 18460747 10.1088/0031-9155/53/11/005

[25] Andreo P , Burns DT , Kapch RP , et al. Determination of consensus k_Q_ values for megavoltage photon beams for the update of IAEA TRS‐398. Phys Med Biol. 2020;65(9):095011. 32182598 10.1088/1361-6560/ab807b

[26] JCGM 100:2008 . Guide to the Expression of Uncertainty in Measurement (Joint Committee for Guides in Metrology, 2008).

